# Women in the field of multiple sclerosis: How they contributed to paradigm shifts

**DOI:** 10.3389/fnmol.2023.1087745

**Published:** 2023-02-03

**Authors:** Andreia Barateiro, Catarina Barros, Maria V. Pinto, Ana Rita Ribeiro, Ainhoa Alberro, Adelaide Fernandes

**Affiliations:** ^1^Central Nervous System, Blood and Peripheral Inflammation Lab, Research Institute for Medicines (iMed.ULisboa), Faculty of Pharmacy, Universidade de Lisboa, Lisbon, Portugal; ^2^Department of Pharmaceutical Sciences and Medicines, Faculty of Pharmacy, Universidade de Lisboa, Lisbon, Portugal; ^3^Multiple Sclerosis Group, Biodonostia Health Research Institute, Donostia-San Sebastian, Spain

**Keywords:** multiple sclerosis, cognitive impairment (CI), immune cell response, peripheral-CNS interplay, white and gray matter damage

## Abstract

History is full of women who made enormous contributions to science. While there is little to no imbalance at the early career stage, a decreasing proportion of women is found as seniority increases. In the multiple sclerosis (MS) field, 44% of first authors and only 35% of senior authors were female. So, in this review, we highlight ground-breaking research done by women in the field of MS, focusing mostly on their work as principal investigators. MS is an autoimmune disorder of the central nervous system (CNS), with evident paradigm shifts in the understating of its pathophysiology. It is known that the immune system becomes overactivated and attacks myelin sheath surrounding axons. The resulting demyelination disrupts the communication signals to and from the CNS, which causes unpredictable symptoms, depending on the neurons that are affected. Classically, MS was reported to cause mostly physical and motor disabilities. However, it is now recognized that cognitive impairment affects more than 50% of the MS patients. Another shifting paradigm was the involvement of gray matter in MS pathology, formerly considered to be a white matter disease. Additionally, the identification of different T cell immune subsets and the mechanisms underlying the involvement of B cells and peripheral macrophages provided a better understanding of the immunopathophysiological processes present in MS. Relevantly, the gut-brain axis, recognized as a bi-directional communication system between the CNS and the gut, was found to be crucial in MS. Indeed, gut microbiota influences not only different susceptibilities to MS pathology, but it can also be modulated in order to positively act in MS course. Also, after the identification of the first microRNA in 1993, the role of microRNAs has been investigated in MS, either as potential biomarkers or therapeutic agents. Finally, concerning MS therapeutical approaches, remyelination-based studies have arisen on the spotlight aiming to repair myelin loss/neuronal connectivity. Altogether, here we emphasize the new insights of remarkable women that have voiced the impact of cognitive impairment, white and gray matter pathology, immune response, and that of the CNS-peripheral interplay on MS diagnosis, progression, and/or therapy efficacy, leading to huge breakthroughs in the MS field.

## 1. Introduction

Although the history of multiple sclerosis (MS) is mostly dominated by breakthroughs of famous men, women have also played a leading role in changing some paradigms that have revolutionized not only the way of thinking about disease onset/progression but also the search for new therapies.

MS is a heterogeneous and unpredictable disease characterized by demyelination, inflammation, and neurodegeneration that affects the central nervous system (CNS) white matter (WM), and gray matter (GM). The formation of demyelinating lesions is a key hallmark of this disease and, depending on the site, symptomatology can range from physical disability to cognitive impairment (CI). This disease affects mainly women (female to male ratio of nearly 3:1) and it is the second cause of neurological disability in young adults between 20 and 40 years of age (Calabrese et al., 2009; Filippi et al., 2018). The clinical symptoms are entirely attributed to CNS pathology and may include changes in visual acuity, motor disturbances, incoordination, bowel and bladder incontinence, and sensory deficits (loss of touch, pain, and temperature). Additionally, other clinical manifestations such as fatigue, dysphagia, psychiatric disorders, and cognitive deficits significantly affect the quality of life of patients and their caregivers (Solaro et al., 2016; Lorefice et al., 2018).

Multiple sclerosis is an immune-mediated disorder, where the etiology cause remains elusive. Indeed, multiple triggering factors have been suggested including genetic and environmental factors, being the most widely investigated the Epstein–Barr virus (EBV) infection, smoking, low levels of vitamin D, obesity, and comorbidities (Amato et al., 2018; Thompson et al., 2018). Specifically, in a recent epidemiology study regarding EBV infection and MS, Bjornevik et al. (2022) found that the risk of MS development increases 32-fold in individuals after infection with this virus. Intriguingly, the risk does not increase with other viruses highlighting that EBV infection is a prerequisite for MS development (Bjornevik et al., 2022). Moreover, in the last decade, the high prevalence of some comorbidities including depression, anxiety and hypertension in MS have been reported (Marrie, 2019). Indeed, comorbidity may influence MS throughout its course, namely, disease severity, relapse rate, patients’ quality of life, treatment efficacy, and mortality (Kowalec et al., 2017; Zhang et al., 2018; Marrie, 2019). In terms of pathology, it is well accepted that in early stages, neural damage arises from T-cell-mediated autoimmunity targeted against the major constituents of the myelin sheath, such as myelin proteolipid protein (PLP). Early in 1986, Marjorie Lees demonstrated that in rabbits, PLP can induce an autoimmune demyelinating disease, the experimental autoimmune encephalomyelitis (EAE), an animal model of MS that displays similar features and pathology to MS (Sobel et al., 1986). Moreover, the different neurological symptoms and signs may reflect the presence and distribution of demyelinating lesions. Patients that have a single episode of neurologic disability, that last at least 24 hours, without dissemination, are usually diagnosed with clinically isolated syndrome (CIS). CIS is now considered part of the MS disease course. On the other hand, incidentally identification of magnetic resonance imaging (MRI) abnormalities in the absence of clinical signs is known as radiologically isolated syndrome (RIS). To avoid misleading diagnostic with other neurological disorders, RIS has to comprise dissemination in space and lesion-specific morphologic features. Although it is not considered a distinct MS phenotype, there is increasing evidence that patients diagnosed with RIS have higher risk of developing definitive MS (Klineova and Lublin, 2018). However, the commonest form of this disease, the relapsing-remitting form of MS (RRMS), involves an initial course, running for several years to more than a decade and is characterized by episodes of relapse (new focal neurologic signs and symptoms caused by inflammation and demyelination) followed by periods of remission or partial recovery. Ultimately, this leads to the formation of multiple CNS lesions and thus, neurological signs. In about one-half of MS cases, the patients develop a progressive form of the disease where insidious disability worsening is independent of clinically apparent relapses (i.e., secondary progressive MS – SPMS). Moreover, in approximately 10% of MS cases, occurs a primarily progressive phase from onset without relapses during the disease known as primary progressive MS (PPMS) (Lublin et al., 2014).

The first putative reported case of MS was identified in a woman, the Saint Lidwina of Schiedam, although no confirmation with brain analysis was performed then. Historical texts reveal that she was affected by a debilitating disease, sharing many characteristics with MS, such as the age of onset, duration, and course of the disease. Briefly, at the age of 16, while ice-skating on a frozen canal, she fell and broke one of her right ribs. Healing was low and, at the age of 19, she began to exhibit other symptoms, including violent pain in her teeth, walking difficulties, sensibility disturbances, blindness in one eye, difficulties in swallowing, and partial paralysis, which may be very well explained by the various WM lesions of the CNS. Moreover, the disease developed very slowly over a period of 37 years, with apparent periods of partial improvement, but generally, the disorder progressed, with symptoms and clinical course typical of MS (Grzegorski and Losy, 2019).

MS is a complex disease whose exact cause is not still known. Given its nature, several paradigms shifted, leading to a crucial transition not only in the understanding of MS as a disease but also in patients’ treatment and management. This review endeavors to highlight the fundamental role of women’s discoveries to break these paradigms, giving relevance to senior authors, since it was found that the proportion of women decreases with seniority. In the MS field, it changes from 44% as first author to 35% as senior author (Thomson et al., 2019). Gender inequality is even higher in terms of authorship of phase 3 clinical trials of drug treatment for MS, where only 23% of authors are women. Moreover, only 16% of the authors named in 3 or more clinical trials are women; only 10% women if it is named in 3 or more clinical trials (not named as first or last author); and only 10% (one woman) if it is named in 6 or more clinical trials (not named as first or last author) (Moneim et al., 2018). Moreover, more recently it was shown that in controlled clinical trials of four monoclonal antibodies used to treat MS (natalizumab, rituximab, alemtuzumab, and ocrelizumab) only 18.2% of the first authors are a woman (Alonso-Moreno et al., 2021). Given that, we believe that improving recognition of female researchers will contribute to changing traditional mindsets and encourage more girls and women to pursue solid science careers.

## 2. Multiple sclerosis symptomatology: Cognitive impairment

Historically, the attention of researchers and clinicians has tended to focus on the physical and motor disabilities of MS patients. At the time, they believed that impaired cognition was not directly correlated with disease onset and progression. However, throughout time, this paradigm started to change with the consequent recognition that cognitive changes are intrinsically associated with disease progression and that preserving cognition is essential for improving patients’ quality of life.

Nowadays is fully accepted that cognitive dysfunction is a core feature of MS, being present since the very beginning of the disease and even in subjects with CIS (Feuillet et al., 2007; Glanz et al., 2007; Potagas et al., 2008). Indeed, cognitive dysfunction is highly variable, being estimated that its prevalence range between 40 and 75% (Amato et al., 2008b; Benedict et al., 2020). In adult-onset patients, the profile of CI shows that visuospatial abilities, memory, complex attention, information processing speed, and executive functions are the most commonly affected domains, whereas semantic memory, attentive span, and language functions are relatively preserved (Bobholz and Rao, 2003; Sumowski et al., 2018; Benedict et al., 2020). Independently of the degree of physical impairment, CI has a high functional impact not only on daily and social life activities but also leads to premature loss of work enhancing the risk of disease progression (Strober et al., 2014; Frndak et al., 2015). Moreover, CI can also limit the capacity of the patient to follow complex treatment regimens and to benefit from inpatient rehabilitation (Langdon and Thompson, 1999).

In the late 80s, some women have started to contribute to shift this paradigm. Elizabeth K. Warrington group demonstrated that cognitive functions as verbal and visual memory, abstracting ability, visual and auditory attention, and naming ability, were significantly worse in patients with CIS when compared with physically disabled controls, being these deficits correlated with the duration of neurological symptoms and with the degree of brain pathology (Callanan et al., 1989). In the same year, Patricia A. Beatty showed that RRMS patients have deficits on tests of information processing speed, verbal fluency, and problem solving, and on recall measures of anterograde and remote memory when compared with control patients (Beatty et al., 1989).

Then, in 1995, Maria Pio Amato first demonstrated that patients with early MS, independently of their work and social activities, presented deficits in verbal memory and abstract reasoning on initial testing that were maintained 4 years later, emerging also at this time linguistic disturbances (Amato et al., 1995). Moreover, in a reassessment 10 years later, she showed that the same patients maintained the previously detected cognitive defects, but also developed deficits in attention/short-term spatial memory (Amato et al., 2001). Later in 2006, Maria Pio Amato revealed for the first time that the prevalence of CI in patients with benign MS [defined as disease duration ±15 years and Expanded Disability Status Scale (EDSS) score ≤ 3.0] was in the range estimated for the general population of MS patients (45%), with prominent involvement of attention, concentration, executive functions and word fluency (Amato et al., 2006). In addition, regarding childhood and juvenile MS, Amato et al. (2008a) found a prevalence of 31% of significant CI and 53% of minor degrees of cognitive dysfunction, being the pattern very similar to those observed in benign MS with the involvement of linguistic abilities. Interestingly, Amato’s group showed that CI is detectable in 57% of patients with CIS, 88% of patients failing three tests had a conversion to MS, which suggests that among clinical variables, CI can serve as an early, sensitive marker of short-term disease evolution, having a prognostic value in predicting conversion to MS (Zipoli et al., 2010). Regarding RRMS, in a controlled and longitudinal study, CI was detected in 30.6% of patients starting therapy with interferon (IFN)-β that worsened in approximately one-third of patients after 3 years, where the presence of moderate impairment at baseline was able to predict further cognitive deterioration (Amato et al., 2010). Moreover, the pattern of cognitive dysfunction also changed over time, involving progressively memory functions, complex attention, and verbal fluency. Furthermore, when comparing the prevalence and profile of CI in older patients with younger ones, she has demonstrated that besides frequency being higher in older patients (77.4%), the profile is similar between them with prominent involvement of information processing speed rather than other domains, which suggest that CI is most directly related to MS itself and not to comorbid age-related disorders (Branco et al., 2019). Recently, it has also been identified different cognitive phenotypes that are a more meaningful measure of the cognitive status of patients and can help to define clinical disability, supporting clinicians in treatment choices, and tailor cognitive rehabilitation strategies to subgroups of cognitively homogeneous patients (De Meo et al., 2021). In the same year, her group also demonstrated that the brain-derived neurotrophic factor (BDNF) and its Val66Met polymorphism may represent a potential biomarker for susceptibility to and severity of CI in MS due to their protective role in cognitive dysfunction (De Meo et al., 2022).

Deficits in cognition associated to MS have no effective treatment yet, being an unmet need to understand the mechanisms underlying this impacting symptom. To fulfill this gap, Maria A. Rocca explored the mechanisms associated with CI, particularly in terms of brain-affected areas. Firstly, in a cohort of patients with PPMS and SPMS diagnosed with CI, she identified a set of brain regions termed default-mode network, including the medial prefrontal cortex, left precentral gyrus, and cingulate cortex, that presents a task-related decrease of intrinsic brain activity across a broad range of cognitive tasks when compared with cognitively preserved MS patients (Rocca et al., 2010b). Furthermore, compared with cognitively impaired PPMS patients, cognitively preserved patients had increased task-related activations on the left caudate nucleus, prefrontal cortex, and inferior parietal lobule (Rocca et al., 2010a). Then, Maria A. Rocca showed that reduced competence in information exchange between distant brain areas contributes to cognitive deficits observed in MS patients since when compared with healthy controls, these patients showed loss of hubs in the superior frontal gyrus, precuneus, and anterior cingulum in the left hemisphere, different lateralization of basal ganglia hubs, and formation of hubs in the left temporal pole and cerebellum (Rocca et al., 2016). Moreover, in the early phases of the disease, there is an involvement of the cerebellar regions that show volumetric abnormalities, particularly in posterior-inferior volume, leading to impaired attention, working memory, and verbal fluency (D’Ambrosio et al., 2017b). Preziosa et al. (2017) also described that impaired cognition in MS is not only due to intrinsic cortical lesion damage but also to severe diffusivity abnormalities in WM lesions and normal-appearing white matter (NAWM). Additionally, CI in MS can also be due to abnormalities of thalamic and cerebellar networks, alongside increased resting-state and functional connectivity between almost all thalamic subregions (frontal, motor, occipital and, most prominently, temporal) and several temporal areas (hippocampus, parahippocampal gyrus, and superior temporal cortex), possibly reflecting a maladaptive mechanism (d’Ambrosio et al., 2017a; Rocca et al., 2018). More recently, Maria A. Rocca group also demonstrated that in patients with benign MS, CI is associated with structural damage of relevant brain areas, such as corpus callosum, posterior corona radiata, and caudate nucleus (Riccitelli et al., 2020).

Several other studies have contributed to this paradigm shift. Back in 1994, Lauren B. Krupp has also demonstrated that 60% of the patients with this disease are cognitively impaired, being these deficits more frequent and widespread when compared to patients with chronic fatigue syndrome (Krupp et al., 1994). They also demonstrated that baseline measures of negative effects, e.g., depressed mood, state of anxiety, and negative affective state, predicted cognitive changes after 1 year in patients with RRMS and SPMS, being higher baseline levels associated with greater relative declines in CI (Christodoulou et al., 2009). Later, Denney et al. (2005) and Bodling et al. (2008) demonstrated that slowing in the speed information processing is a key feature of MS patients, being observed in the different subtypes. Brissart et al. (2013) have also described different cognitive profiles in MS, where, verbal fluency is impaired at an early stage of RRMS, and episodic memory, working memory, flexibility, semantic, and phonemic fluencies are affected in progressive forms. Carotenuto et al. (2019), in a retrospective multicenter study including RRMS patients with an early onset of disease, have shown that worse cognitive function in specific domains (visual memory and attention/information processing) at disease onset is associated with increased risk of relapses and of sustained disability progression up to 7 years. Furthermore, Matilde Inglese’s group has shown that CI is frequent in benign MS patients (minimal disability even decades after the onset of symptoms) (38%) and is related to micro-structural damage of WM tracts connecting the brain cortical and sub-cortical regions of the two hemispheres (Bester et al., 2013). Iris-Katharina Penner demonstrated that WM lesions (Papadopoulou et al., 2013), corpus callosum atrophy (Yaldizli et al., 2014), and cerebellar abnormalities (Weier et al., 2014) have a crucial role in the development of cognitive deficits, especially attention and information-processing speed.

## 3. Multiple sclerosis brain pathology: Gray matter

MS has historically been classified as a WM disease because of the inflammation and demyelination occurring in this CNS region. However, the pathological observations in the WM did not always explain or predict the clinical symptoms observed in patients with MS. In more recent years, several studies have shown not only that GM is also affected, but also that its atrophy is a clinically relevant component of the overall brain atrophy that can be associated with disability in MS (Chard et al., 2004; Tedeschi et al., 2005).

The first study to demonstrate that MS is related to GM lesions was from Isabelle Catalaa that in 1999 found that cortical and deep GM lesions comprised approximately 5.7 and 4.6%, respectively, of the total lesion volume in patients with RRMS (Catalaa et al., 1999). Since 2009, Olga Ciccarelli has also developed an extensive work on the impact of GM in MS pathology. In patients with PPMS, she described pathological processes occurring in the NAWM and in the GM in specific, clinically relevant brain areas, such as the right sensory-motor cortex and the connected corticospinal tract, both thalami and the adjacent thalamic radiations, and the left insula and the adjacent WM (Bodini et al., 2009). Later, she also demonstrated that GM damage, which reflects axonal loss, is strongly correlated with overall cognitive dysfunction in patients with PPMS, emphasizing the impact of abnormal GM integrity in MS (Tur et al., 2011). Interestingly, patients with PPMS showed over 5-years, a greater decline of GM volume bilaterally in the cingulate cortex, thalamus, putamen, precentral gyrus, insula, and cerebellum when compared with healthy controls. Additionally, they observed volume loss in the left insula, left precuneus, right cingulate cortex, bilateral putamen, and left superior temporal gyrus when compared to baseline, which occurs at different speed rates across these regions (Eshaghi et al., 2014). The following studies from Ciccarelli’s group aimed to understand which of the GM regions become atrophic and their association with disability accumulation. Particularly, in the initial phases, as CIS and RRMS, the first atrophic regions are the posterior cingulate cortex, precuneus cerebellum, caudate, and putamen, followed by the middle cingulated cortex, brainstem, and thalamus. Furthermore, in PPMS, there is the involvement of the thalamus, cuneus, precuneus, and pallidum, followed by the brainstem, posterior cingulate cortex, cerebellum, caudate, and putamen (Eshaghi et al., 2018a). In a multicenter and longitudinal study, they have also demonstrated that over time cortical GM atrophy, particularly in temporal and parietal lobes, was faster when compared to other brain regions, driving disability accumulation in MS (Eshaghi et al., 2018b). Recently, Maria A. Rocca and Olga Ciccarelli have shown that in 1-year, GM atrophy differs across disease phenotypes and progresses in MS subcortical, cerebellar, sensorimotor, and fronto-temporo-parietal components. They highlight that sensorimotor and cerebellar GM atrophy could explain baseline disability and clinical worsening (Rocca et al., 2021). From a pathological point of view, Bodini et al. (2016) demonstrated that in early PPMS occurs a relationship between anatomically GM and WM pathology, with cortical damage mostly being a sequela of NAWM pathology (Bodini et al., 2016). Concerning the mechanisms involved in the appearance of GM lesions, Muhlert et al. (2014) have shown a positive relationship between glutamate levels in the hippocampus, thalamus, and cingulate region and visuospatial memory in patients, which is not observed in healthy controls, suggesting the reliance of memory on glutamatergic systems. In this sense, also sodium accumulation in cortical GM was shown to reflect underlying neuroaxonal metabolic abnormalities relevant to disease course heterogeneity and disability in RRMS (Brownlee et al., 2019). Additionally, Matilde Inglese’s group demonstrated that global GM and WM total sodium concentration, and intracellular sodium concentration were higher while GM and WM intracellular sodium volume fraction (indirect measure of extracellular sodium concentration) were lower in MS patients compared with healthy controls. Moreover, at a brain regional level, clusters of increased total sodium concentration and intracellular sodium concentration and decreased intracellular sodium volume fraction were found in several cortical, subcortical and WM regions (Petracca et al., 2016). Other studies such as the ones led by Kerstin Bendfelt have also contributed to unveiling the involvement of specific GM regions in MS. Indeed, they first showed that patients with RRMS have a significant GM reduction at the level of the anterior and posterior cingulate, the temporal cortex, and the cerebellum, as a consequence of regional GM volume atrophy, which in turn is also associated with WM lesions progression such as in fronto-temporal cortical areas (Bendfeldt et al., 2009). Later, they also observed that significant WM lesion changes were accompanied by cortical GM volume reductions in the corpus callosum and optic radiation in patients with RRMS, suggesting that disconnections between different areas of cortical networks could be related to widespread cortical atrophy and CI (Bendfeldt et al., 2010). Moreover, also in RRMS, longitudinal GM volume reductions were observed bilaterally in the cerebellum, uncus, inferior orbital gyrus, paracentral lobule, precuneus, fronto-temporal, parietal, limbic, and occipital cortical regions of both hemispheres, as well as in the left claustrum (Bendfeldt et al., 2012a). Further studies revealed regional GM volume reductions as in the right parietal lobe, in the right precuneus, and in the right postcentral gyrus, that can be associated with disability progression (Hofstetter et al., 2014). Interestingly, Bendfeldt et al. (2012b) also demonstrated that neuroanatomical spatial patterns of GM segmentations contain enough information for the correct classification of the patients with MS into different groups, such as patients with either early or late MS (contrast I), low or high WM lesion load (contrast II), and benign or non-benign MS (contrast III), being the most relevant patterns the ones that comprise cortical areas of all the cerebral lobes, as well as deep GM structures, including thalamus and caudate.

Many other women have actively contributed to this field. Inglese et al. (2004) presented indirect evidences that RRMS was not exclusively a WM disease and proposed a reclassification as a “diffuse whole-brain disorder”. She also demonstrated that the amount of damage in deep GM structures including the thalamus, putamen, and caudate is correlated with the number of cortical lesions in PPMS (Ruggieri et al., 2015). Another study demonstrated that cortical and subcortical GM injury may play a different role in CI development depending on the disease course. Indeed, in RRMS and PPMS cognitive dysfunction is associated with cortical and subcortical GM injury, respectively (Bester et al., 2015). Later, in 2016, Caterina Mainero’s group demonstrated that cortical changes are related with CI, independently of WM lesions and cortical atrophy. In fact, they found a strong correlation between overall dysfunction and longer MRI changes in several cortical areas that strikingly overlap with regions of the default-mode network, known to be highly critical for attention and memory (e.g., precuneus/posterior cingulate cortex that forms a major hub, medial prefrontal cortex, medial temporal lobe, and lateral and inferior parietal cortex) (Louapre et al., 2016). It was shown that GM loss in the thalamus and basal ganglia is associated with worse cognition in MS (Cruz-Gomez et al., 2018). Furthermore, Cristina Forn reported that GM atrophy is circumscribed to the bilateral posterior cingulate gyrus/precuneus in patients with early MS (Baltruschat et al., 2015). Interestingly, GM volume appears to differ concerning sex. Specifically, male MS patients showed a higher degree of GM volume in the bilateral frontal areas and increased functional connectivity as a compensatory mechanism when compared with female patients (Sanchis-Segura et al., 2016). Moreover, in another study, it was shown that GM loss in the thalamus and basal ganglia was associated with worse cognition in MS (Cruz-Gomez et al., 2018).

Concerning the mechanisms underlying GM atrophy, Anne-Marie Van Dam started to demonstrate, in chronic-relapsing EAE rats, that in early clinical phases of experimental MS, interleukin (IL)-1β and IL-1 receptor antagonist are expressed, mainly by macrophages and/or microglia, in several brain and spinal cord regions of GM and WM lesions, particularly in perivascular and periventricular locations. In fact, this contributed to the development of GM lesions and explained various clinical deficits present in MS (Prins et al., 2013). Moreover, it is also known that transglutaminase, in GM lesions, is mainly present in microglial cells that express β1-integrin, suggesting its involvement in microglia proliferation (Espitia Pinzon et al., 2014). Moreover, Prins et al. (2014) demonstrated for the first time that there is a spatial and quantitative discrepancy in the distribution of CCL2 and CCR2 in the hippocampus, being both detected in hippocampal WM lesions whereas only CCR2 is present in GM lesions, possibly explaining the lack of infiltrating immune cells in these lesions. Recent findings showed that neighboring WM and GM lesions have a distinct cellular composition and gene expression profile. Particularly in GM, there was higher expression of clusters involved in immune response and cellular activation, suggesting that the pathological processes underlying demyelination in both GM and WM lesions are different (van Wageningen et al., 2022). On the other hand, Vercellino et al. (2005, 2009, 2022) showed that neuronal and synaptic density were significantly reduced in GM and deep GM lesions hypothesizing a substantial effect of GM on clinical disability and cognitive deficits.

## 4. The immune system in multiple sclerosis

The immune system plays a key role in MS pathology contributing to neuroinflammation, demyelination, and neuroaxonal degeneration, ultimately leading to the formation of lesions. Throughout time, the identification of immune cell subsets has provided a paradigm for understanding multiple pathophysiological processes of MS. Among the effector CD4 T cells subsets, T helper (Th) cells as Th1, Th2, and Th17 cells, and regulatory T cells (Tregs) rapidly were found to be implicated in the disease. Several works conducted by Estelle Bettelli aimed to study the pathways underlying the effects of these cells. Her first studies, using the EAE model, found that mice lacking T-bet, an important transcription factor, were protected from EAE development and failed to generate Th1 cells, suggesting a key role of this transcription factor in regulating Th1 cells in EAE (Bettelli et al., 2004). Then, she also demonstrated that there is a reciprocal developmental pathway for the generation of pathogenic Th17 cells and protective Treg cells depending on the production of specific cytokines like transforming growth factor β (TGFβ) and IL-6 (Bettelli et al., 2006). Afterward, Peters et al. (2011) found that, in the EAE adoptive transfer of Th17 cells, the development of ectopic lymphoid follicles was partially dependent on Th17-specific cytokines as IL-17 and on the cell surface molecule podoplanin highly expressed by these cells. Following these studies, as group leader, Estelle Bettelli’s lab unveiled a novel mechanism by which Th17 cells can be converted to IFNγ-producing Th1 cells in response to chronic IL-7 stimulation in the CNS during EAE (Arbelaez et al., 2015). Furthermore, after isolation of T cells from autologous cerebrospinal fluid (CSF) of patients with MS, Planas et al. (2015) provided the first functional evidence for the putative role of Th2 cells in specific MS lesions (Pattern II) supporting the existence of their pathogenic phenotype. Furthermore, an important aspect regarding MS pathology is the lymphocyte migration to brain parenchyma, being a crucial step to initiate tissue inflammation and damage in MS. However, the molecular processes needed for that to occur are poorly understood. Reboldi et al. (2009) hypothesized that T cells migration occurs in two waves. While the first wave of effector cells migration occurs via a CCR6-dependent way through epithelial cells of the choroid plexus, the second wave occurs via a CCR6-independent way across the blood-brain barrier (Reboldi et al., 2009). In addition to the importance of effector T cells and their migration, the identification of autoantigens in MS can be essential for the development of new therapeutic strategies. By analyzing CSF from patients with MS, Cruciani et al. (2021) identified GDP-L-fucose synthase responders with a significant expansion of an effector memory Th1 subset. Further studies from the same group also aimed to study the involvement of antigen-induced T cell senescence in controlling CD4 T cell-mediated responses in MS. Indeed, high levels of CD4 T cells showed increased frequencies of costimulatory molecules with proinflammatory Th1 phenotypes and positively correlate with the levels of neurofilament light chain (Tomas-Ojer et al., 2022). Together, these studies highlight the role of effector T cells including Th1, Th2, and Th17 cells in MS pathogenesis, shed some light on ways of lymphocytes entrance into the CNS, and strongly supported the importance of MS antigens in T cell specificity and senescence.

Although CD4 T cells have been established as important players, CD8 T cells are increasingly recognized as potential contributors to tissue damage being outnumber in MS lesions. Nathalie Arbour focused on the impact of programmed cell death protein 1 (PD-1), a co-inhibitory receptor, on CD8 T cell functions in MS. Although they found that PD-1 ligand (PD-L1) has high ability to modulate CD8 T cells, this does not happen in MS conditions. Their data suggest that, during MS pathogenesis, the inflamed CNS attempts to protect itself via PD-L1. However, they fail to do it because CD8 T cells express low amounts of its receptor and consequently are not able to produce a co-inhibitory stimulus (Pittet et al., 2011a). Based on this study, Nathalie Arbour’s group further demonstrated that brain endothelial cells contribute to control CD8 T cell transmigration into the CNS due to the upregulation of PD-1 ligands like PD-L2. Interestingly, in MS, this mechanism is impaired possibly allowing the uncontrolled migration of cytotoxic cells (Pittet et al., 2011b).

Although it is well-accepted that MS is a T cell-mediated disease, there has been a dramatic shift regarding the additional involvement of other immune cells, particularly B cells. Indeed, in 1999, the group led by Anne H. Cross sought to solve this issue and offered new perspectives by inducing EAE in B cell-deficient mice using either MOG35-55 protein or a recombinant MOG (rMOG) peptide on C56BL/6 mice strain. Interestingly, while wild-type (WT) mice were readily susceptible to rMOG-induced disease developing a normal disease course, B cell-deficient mice failed to show clinical symptoms. Moreover, histological data also showed that animals immunized with rMOG had significantly less inflammation, demyelination, and axonal loss. With these results, Lyons et al. (1999) were the first group to demonstrate that B cells are required to induce EAE and further hypothesized that the lack of pathology might be due to the absence of antibodies in B cell-deficient mice. In subsequent studies, they also found that passive transfer of activated CD19+ B cells was able to reconstitute the ability to develop clinical symptoms, although with later disease onset and less severity when compared to WT mice. Then, to investigate whether the role of rMOG antibodies was crucial in priming encephalitogenic T cells or in later disease pathogenesis, B cell-deficient mice previously immunized were treated with rMOG-primed serum. They observed a rapid appearance of clinical signs and CNS inflammation highlighting the critical role of MOG-specific antibodies in the initiation of murine EAE (Lyons et al., 2002). Further studies also characterized the role of B cells in the pathogenesis of EAE using another myelin peptide, the PLP, on the BALB/c strain. Contrary to what they previously observed with MOG induction, here they showed severe EAE symptoms in the absence of B cells, highlighting the differences in peptide-induced EAE and genetic differences between mouse strains (Lyons et al., 2008). More recently, Nancy L. Monson’s group demonstrated that B cell depletion using CD19 monoclonal antibody (mAb) treatment suppresses EAE clinical course when induced with the recombinant human MOG1-125 (rhMOG1-125). Additionally, it inhibited pathogenic adaptive immune responses and preserved peripheral regulatory mechanisms of regulatory B cells and antigen T cell responses. Overall, the data suggest that CD19 mAb may represent a new approach for depleting B cells and plasma cells by inhibiting the generation of new myelin-specific antibodies (Chen et al., 2016). Recently, Wang et al. (2021) reviewed the role of plasma cells in both animal and human pathology. Indeed, they described their importance as a continuous source of antibody production and showed their regulatory role by producing IL-10 and IL-35 (Wang et al., 2021). Afterward, Pierson et al. (2014) addressed the role of B cells within CNS during early T cell infiltration in their developed animal model of EAE in C3HeB/Fej (B cell-deficient) mice. As the group led by Anne H. Cross, they observed that B cell-deficient mice have less EAE incidence than WT mice, highlighting the critical role of B cells in initiating inflammatory responses. In the absence of B cells, adoptive transfer of T cells showed initial infiltration into the CNS, although they were not able to trigger the inflammatory response needed to recruit cells from the periphery. *In vitro* studies also revealed that B cells preferentially require exogenous IL-1β to reactivate Th17 cells. This study identifies a crucial role of B cells in T cell reactivation within the CNS corroborating their influence in disease development (Pierson et al., 2014). Additional studies from Nancy L. Monson’s lab strengthened these hypotheses, by demonstrating that B cells from MS patients can support Th17 responses through stimulation of IL-17 cytokine production in response to neuro-antigens (Ireland et al., 2016).

The germinal centers of lymphoid follicles are the microenvironment where activated B cells undergo clonal expansion and selection to differentiate into memory B cells or into plasma cells secreting antibodies. Given the importance of these structures, Francesca Aloisi, aimed to identify them in *postmortem* brain and spinal cord tissue from patients with MS within different disease courses (RRMS, PPMS, and SPMS). Indeed, they showed for the first time the presence of lymphoid follicle-like structures containing B cells only in the meninges of patients with SPMS. Also, they found that B cells were in a proliferative state, strongly suggestive of germinal center formation and possibly contributing to compartmentalized humoral immune response and thus disease exacerbation (Serafini et al., 2004). To further understand how the presence of ectopic B cell follicles in MS could be associated with clinical course and pathological features, Magliozzi et al. (2007) performed a detailed analysis using *postmortem* brain tissues of patients with MS. Interestingly, they were able to find that the presence of ectopic B cell follicles could anticipate the time of clinical onset and the presence of these structures correlated with a higher number of cortical demyelinating lesions exacerbating cortical damage and contributing to GM pathology (Magliozzi et al., 2007). Expanding on previous findings, recent studies conducted by Olga Ciccarelli’s group aimed to correlate perivascular B cell infiltrates with MS clinical severity. They reported that patients with progressive forms of MS with the presence of compartmentalized perivascular CD20 B lymphocytes *postmortem* had an earlier and more severe onset, and higher rates of death (Moccia et al., 2022).

Evidence supporting the role of B cells in the pathogenesis of MS, not only in the murine animal model but also in humans, rapidly turned these cells into the most studied therapeutic target. The approaches of selective B cell-depleting therapies opened new doors for the treatment of MS and already demonstrated the strong efficacy and safety. Recently, Margoni et al. (2022) summarized the most relevant findings supporting B cell involvement in MS and highlighted anti-CD20 therapies. Until now, there are three approved anti-CD20 monoclonal antibodies: the chimeric mouse antibody – rituximab; the humanized antibody – ocrelizumab; and the fully human antibody – ofatumumab. Hopefully, ublituximab, a chimeric mouse human antibody that is now in Phase III clinical trials, will be the next anti-CD20 monoclonal antibody added to the MS treatment options (Margoni et al., 2022).

Additionally, many studies have highlighted the contribution of peripheral macrophages to this peripheral-CNS interplay in the context of MS. It is known that macrophages of the periphery and monocyte-derived ones are recruited to CNS lesions in response to chemokine signaling. The role of peripheral macrophages in MS was initially considered to be predominantly neuro-destructive, leading to myelin destruction and to axonal damage. Accordingly, in Athena Soulika’s lab, EAE-induced mice were injected with clodronate liposomes, right after the appearance of the first clinical symptoms, to induce the apoptosis of infiltrating monocyte-derived cells in the blood and spleen. By doing so, they observed that prolonged depletion of infiltrating macrophages was able to ameliorate the severity of the EAE-acquired clinical symptoms and to reduce the myelin loss and axonal injury at the peak of EAE (Moreno et al., 2016). In line with this, Lydia Sorokin’s group additionally showed that resident and infiltrating macrophages express sialoadhesin (Sn), a sialic acid-binding transmembrane protein that directly interacts with Sn ligands on Tregs. In this study, the authors observed that macrophage Sn-Tregs interaction negatively impacted Treg proliferation capacity *in vitro*, and *in vivo* using Sn-knockout mice. Indeed, these EAE-induced Sn knockout animals showed reduced severity of the symptoms and decreased susceptibility to EAE, highlighting the relevance of the macrophage population on T cell balance and, consequently, on disease progression (Wu et al., 2009).

However, macrophages’ role in MS is rather complex given their highly heterogeneous functionalities. Apart from their above-mentioned detrimental roles in MS-related models, other studies revealed that infiltrating macrophages can have intermediate phenotypes or even opposed tissue-repairing functions. Indeed, Christine Dijkstra’s group described not only the presence of both M1 and M2-like macrophages in active MS lesions but also the presence of a population of perivascular macrophages with an intermediate phenotype, co-expressing both CD40 and the mannose receptor (CD206) (Vogel et al., 2013). More importantly, Tierney et al. (2009) demonstrated that type-II-activated macrophages with anti-inflammatory properties, obtained by exposure to immune complexes such as the opsonized sheep red blood cells, have a major role in the EAE pathology. Here, the authors revealed that type-II-activated macrophage injection 4 hours before EAE induction, prevented mice from developing EAE-associated clinical symptoms (Tierney et al., 2009). Relevantly, in the same line of research, using IL-4-induced alternative activated bone marrow-derived macrophages, with immunosuppressive functions, Michal Schwartz’s lab also demonstrated that these macrophages had a suppressive effect on the EAE-associated clinical features that were not observed in the non-injected EAE mice, thus corroborating the protective role of M2-like macrophages in MS-based models, and overall confirming the major contribution of different peripheral cells and phenotypes to either MS onset, progression, and recovery (Vaknin et al., 2011).

## 5. Gut-brain axis in multiple sclerosis

In the last decades, the gut-brain axis, which is defined as bidirectional communication between the CNS and the intestine, has been linked to a variety of neurodegenerative disorders including MS. Indeed, the first studies emerged in the 90s from Joan Goverman’s pioneering work using a transgenic mouse of spontaneous EAE (Goverman et al., 1993). Here, they found that mice housed in a non-sterilized environment can develop spontaneous EAE but not those housed in a germ-free controlled environment. Her study opened a new door to understand the influence of environmental factors on the incidence of spontaneous EAE. Based on this work, Brabb et al. (1997) used the same transgenic mouse model under controlled conditions to eliminate possible environmental variables. They described that the incidence of EAE in transgenic mice with conventional microbial flora was 43% but for the specific pathogen-free mouse was only 15%. Moreover, these two studies were the first to mention that spontaneous EAE could result from changes in the immune system due to generalized microbial stimulation. In line with the interaction between the immune system and gut microbiome, in 2017, the group led by Mitzi Nagarkatti published a study addressing the impact of CD44 deletion in EAE and its effects on gut microbial composition. They found that host genetics induce differences in the immune responses and affect gut composition, which is important for EAE and MS pathophysiology (Chitrala et al., 2017). Recently, Jennifer L. Gommerman’s group published a study on the role of gut derived IgA^+^ B cells on CNS inflammation in EAE model. They found that gut-derived IgA^+^ B cells are able to access CNS in EAE mice and suppress inflammation, thus attenuating disease progression with production of anti-inflammatory cytokines. Importantly, this study emerged to reconsider plasma cells’ role during MS and the potential to use IgA^+^ B cells as an immunosuppressive approach to attenuate MS (Rojas et al., 2019).

After first insight into the influence of microbiome in EAE, Nagarkatti’s group published another study in 2019 showing the role of gut microbiota in the development of different forms of MS. For that, they used two different animal models: C57BL/6 mice (mimics a chronic-progressive course of MS); and SJL/J mice (mimics a relapsing-remitting RR-EAE course of the disease). First, they found a significantly diverse microbial composition in the two strains before EAE induction that could be used as a predictor biomarker and as an indicator of the type of disease course. Interestingly, not only naïve strains presented distinct abundances of bacteria, but also after EAE induction mice displayed differences in microbial composition. To conclude, this study was an open door into the impact of the gut-brain axis in MS and allowed the identification of different gut microbial compositions that may shape and contribute to the different EAE courses (Gandy et al., 2019). Together, these studies contributed to highlight the role of gut microbiota in different forms of EAE, which directly influence susceptibilities to and progression of the disease.

Recently, Camen Espejo and her team looked for the therapeutic effects of a mixture containing 17 different strains of *Clostridia*, already reported as being decreased in MS patients, from the human microbiota on the EAE model. For that, EAE mice were treated daily after the first symptoms of the disease. They found that oral administration improved clinical outcomes of EAE together with decreased CNS demyelination and astrogliosis. Transcriptomic analysis revealed an upregulation of IFN-β, which is used as a first-line treatment in MS and ameliorates EAE pathogenesis. More importantly, the authors found increased levels of butyrate, a short-chain fatty acid, in the serum of treated mice that may act as an anti-inflammatory agent at the periphery (Calvo-Barreiro et al., 2021). Considering the outstanding results, this was one of the few preclinical studies using human-isolated bacteria in the EAE model and the first one to highlight the therapeutic potential of commensal bacteria in rebalancing gut dysbiosis in MS patients. Nevertheless, Carmen Espejo has also been focused on the potential therapeutic use of probiotics. Indeed, she investigated the effects of commercially available probiotics (Lactibiane iki and Vivomixx) using the EAE model. She found that Lactibiane iki, composed of two probiotic strains of *Lactobacillus* and *Bifidobacterium*, was able to regulate immune response and CNS inflammation and demyelination, reverting clinical symptoms in EAE mice (Calvo-Barreiro et al., 2020). Importantly, this study reinforces the potential use of commercially available probiotics to attenuate MS effects on MS patients.

Studies involving MS patients suggest that there are differences in gut composition between patients and healthy controls, as well as along distinct diseases courses as described above. The first data came from the work of Sachiko Miyake. In this study, Miyake aimed to investigate whether gut microbiome in patients with RRMS is altered compared to healthy individuals. Results revealed microbiome dysbiosis in patients with MS with differential relative abundance of bacteria, especially for species belonging *Clostridia clusters XIVa* and *IV* and *Bacteroidetes*. This study highlighted the potential of correcting dysbiosis as a therapeutical approach to prevent and treat MS (Miyake et al., 2015). Moreover, in 2021, Laura Cox investigated gut microbiota in patients with RRMS and with progressive forms of MS and compared them with healthy controls. She found that gut microbiota composition of MS patients differs from healthy controls and MS patients. In progressive MS patients, it was found a unique signature of *Enterobacteriaceae* and *Clostridium g24 FCEY* and decreased *Blautia* and *Agathobaculum* associated with higher degree of disease (Cox et al., 2021). In a very recent study, iMSMSConsortium (2022) studied the microbiota composition differences between untreated MS patients and genetically unrelated household healthy controls of a large cohort sample. They found that microbiota composition and associated functions was significantly altered across disease subtypes, in response to different MS treatments and diet (iMSMSConsortium, 2022).

Studies involving pediatric-onset patients have also been developed in this field. Helen Tremlett and Emmanuelle Wauban recently explored the differences between pediatric-onset MS [with or without exposure to disease-modifying drugs (DMDs)] and either monophasic acquired demyelinating syndromes patients or unaffected controls. They found differences between groups at the taxa and gut-community-network levels. Specifically, patients with MS (with or without DMD) exhibited a higher abundance of opportunistic pathogens, where *Ruminococcaceae* and *Christensenellaceae*, markers of gut health, were lower in pediatric-onset MS versus unaffected controls (Tremlett et al., 2021). Also, in 2021, Mary Horton from Emmanuele Waubant’s group, observed that in a longitudinal study including pediatric-onset MS patients, a lower hazard of clinical relapse and MRI-related disease activity was associated with differential abundance of butyrate gut microbes’ producers (Horton et al., 2021). These recent studies are extremely important showing that early in life children/adolescents display differences in their gut microbiome flora and associated metabolites that may contribute to MS progression and highlight a potential gut microbiota intervention to modify disease activity. Expanding these results, in 2022, by metagenomic sequencing, they showed differences in the microbiome’s metabolic potential in pediatric-onset MS patients versus unaffected controls. Interestingly, they observed that MS patients exhibited a higher potential to generate lipopolysaccharide and a lower potential to metabolize peptidoglycan and starch compared to unaffected controls. These findings suggest that patients with MS suffer from disturbances in cell-wall and carbohydrate metabolism from their gut microbial community, which might contribute to MS pathology (Mirza et al., 2022).

## 6. MicroRNAs in multiple sclerosis

MicroRNAs (miRNAs) are the most widely studied non-coding RNAs, playing a role in nearly all physiological and pathological processes (Bartel, 2018). They were first identified in the 1990s (Lee et al., 1993) and the translational repression mediated by miRNAs has been investigated by many researchers, also in the MS field since 2009 (Otaegui et al., 2009; Piket et al., 2019).

In 2010, Candace L. Kerr was the first to show that miRNA profiles change during oligodendrocyte (OL) maturation, consequently affecting myelin production (Letzen et al., 2010). Then, Raija L. P. Lindberg studied the expression of miRNAs in CD4+ and CD8+ T cells, as well as in B cells in untreated patients with RRMS and controls. They focused on CD4+ T cells and on miR-17-5p upregulation, and also performed some functional experiments *in vitro* (Sievers et al., 2012). Following this study, Lindberg’s group demonstrated that the expression levels of miR-17 and miR-126 are increased in CD4+ T cells during relapse while being reduced in patients under natalizumab treatment (Sievers et al., 2012; Meira et al., 2014a,b). In a different approach, but also focusing on CD4+ T cells, the group of Amy E. Lovett-Racke found that miR-128 and miR-27b were increased in naïve and miR-340 in memory CD4+ T cells from MS patients. Notably, these three miRNAs inhibited Th2 cells and favored proinflammatory Th1 responses exacerbating disease progression in the EAE model (Guerau-de-Arellano et al., 2011). Lovett-Racke’s group also found that 19 out of the 85 miRNAs differentially expressed in naïve CD4+ T cells were predicted to target the TGFβ signaling pathway, which is critical for Treg development (Severin et al., 2016). In a preliminary study, Lovett-Racke’s group found, in *postmortem* samples, 14 decreased and 1 increased miRNA in NAWM of MS donors compared to healthy controls, where the most significant upregulated and downregulated miRNAs were miR-223 and miR-191, respectively (Guerau-de-Arellano et al., 2015).

The group led by Rosanna Asselta was also one of the first to investigate miRNAs in MS, and to report in 2011 the elevated expression of miR-155, an important regulator of inflammation, in peripheral blood mononuclear cells (PBMCs) from patients with MS compared to controls (Paraboschi et al., 2011). Gandhi et al. (2013) described miRNA differences in plasma samples of patients with RRMS and SPMS, and healthy controls. Interestingly, miR-92a-1-5p was the one differentially expressed between RRMS and SPMS and was also significantly associated with EDSS score and disease duration. Next, Gandhi focused on serum miRNAs in MS, and proposed several miRNA biomarkers that could help the diagnosis and monitoring of MS. She highlighted that miR-337-3p was the most significant miRNA that negatively correlated with the EDSS score and that miR-484 expression differences were the most robust ones between MS and control donors (Regev et al., 2016; Regev et al., 2018).

miRNAs have also been investigated in the EAE model. Comprehensive profiling of miRNAs in EAE was already published in 2013 by two distinct groups, one from Mitzi Nagarkatti and one from Maja Jagodic. In Nagarkatti’s work, authors found that *in vivo* silencing of let-7e attenuated the EAE course in mice and inhibited Th1 and Th17 cells, while the overexpression of let-7e resulted in a more severe EAE (Guan et al., 2013). Jagodic’s work reported differentially expressed miRNAs in draining lymph nodes, including the increased expression of let-7e and other relevant miRNAs, such as miR-146a, miR-21, miR-181a, and miR-223, in EAE animals (Bergman et al., 2013). In the last 10 years, many authors have continued investigating the implications of miRNAs in EAE, but still, further investigations are needed to better understand the complex regulation by miRNAs in the EAE model and in MS. Apart from their work in the EAE model, Jagodic’s group proposed miR-150 in cell-free CSF as an interesting early biomarker of MS. They found elevated levels of miR-150 in MS when compared to inflammatory and non-inflammatory neurologic diseases, and also in patients with CIS who converted to MS compared to those CIS who never converted (Bergman et al., 2016). Additionally, corroborating Jagodic results’ in EAE, Cantoni et al. (2017) found that miR-223^–/–^ animals had a significantly less severe EAE course and more effective myeloid-derived suppressor cells than WT littermates. In contrast, a work coordinated by Alyson E. Fournier reported that miR-223 is upregulated in lesions of MS patients and in motor neurons of EAE mice. Furthermore, the introduction of miR-223 retinal ganglion cells protected axons from degeneration in EAE mice. miR-223 is upregulated in response to inflammation and mediates a compensatory neuroprotective response (Morquette et al., 2019). These works show the complex regulatory effects of a particular miRNA and the diverse effects it can exert in different cell types.

Lastly, the relevance of miRNAs carried by extracellular vesicles (EVs) should be noted. Numerous works have investigated the role and potential therapeutic use of miRNAs loaded in EVs (Alberro et al., 2021). Related to MS, the group of Claudia Verderio performed *in vitro* and *in vivo* studies that demonstrated a glia-to-neuron transfer of miRNAs via EVs. Moreover, they showed that EVs from inflammatory microglia are enriched in miR-146a-5p compared to EVs from pro-regenerative microglia and that the administration of EVs from inflammatory microglia, but not from pro-regenerative microglia, promoted dendritic spine loss in rat hippocampus. Verderio’s group suggested a possible link between microglia activation, enhanced EV production, dendritic spine loss, and cognitive symptoms in patients with MS (Prada et al., 2018). A later work done by the same group evaluated miRNAs in myeloid-EVs from plasma samples of cognitively preserved and cognitively impaired MS donors. They found that myeloid EVs from cognitively impaired patients have higher levels of miR-150-5p and lower levels of let-7b-5p compared to cognitively preserved patients in two independent cohorts, and therefore proposed them as potentially useful biomarkers for MS cognitive impairment (Scaroni et al., 2022).

Similarly, women led important works about other non-coding RNAs and epigenetic signatures in MS. Maja Jagodic’s group recently published a comprehensive profiling of small non-coding RNAs in matching samples from different origins (PBMCs, plasma, CSF cells, and cell-free CSF) from RRMS, SPMS, and inflammatory and non-inflammatory neurologic disease controls (Zheleznyakova et al., 2021). Remarkably, the first work about DNA methylation changes in MS patients was published by the group of Patrizia Casaccia (Huynh et al., 2014) and a relevant report of Jagodic’s group identified a differentially methylated region in HLA-DRB1 and a protective variant (rs9267649) that was associated with increased DNA methylation and lower HLA-DRB1 expression in MS (Kular et al., 2018). In addition, Rosanna Asselta’s group was the first to find a dysregulated circular RNA (circRNA) in MS in 2017. They found a circRNA (hsa_circ_0106803) that is upregulated in PBMCs of RRMS patients when compared to controls and, since then, many works have found differentially expressed circRNAs in MS (Cardamone et al., 2017).

## 7. Remyelination ability during multiple sclerosis

Myelin loss, or demyelination, is also an important hallmark of MS and the major cause of MS-associated deficits in neuronal communication and axonal viability (Lubetzki and Stankoff, 2014). Nowadays, myelin repair-based therapeutics represent a strong and active field in MS research, as it is believed that reverting progressive disability in MS can only be possible through myelin re-ensheathment of denuded axons, thus depending on the extent of remyelinating processes happening post-CNS demyelination.

Remyelination is an endogenous, but challenging, process where new myelin sheaths are generated around demyelinated axons, and the way to reestablish neurological function and provide neuroprotection against further degeneration (Chari, 2007). Endogenous remyelination in a mammalian CNS was first described by Mary Bunge in the 1960s (Bunge et al., 1961) and then in multiple MS lesion samples (Barkhof et al., 2003). Importantly, Benedetta Bodini and her group are known for their multiple studies on the *in vivo* imaging of myelin content changes in MS lesions. Using combined magnetization transfer imaging and positron emission tomography, Bodini’s group demonstrated in 2022 that the extent of myelin loss and repair was highly heterogeneous between white matter and cortical lesions of MS patients. Relevantly they showed that this detected variability could correlate with the differences observed in the clinical scores, increasing the potential of these *in vivo* imaging techniques to be used as good outcome indicators on clinical trials (Lazzarotto et al., 2022). In the same year, using positron emission tomography (PET) combined, this time, with MRI, the group further unveils that the occurrence of remyelination not only provides intralesional axonal protection but also prevents further neuronal degeneration on distal surrounding areas, confirming, *in vivo*, the protective effect of remyelination on MS progression over time (Ricigliano et al., 2022). However, over time, this endogenous recovery becomes consistently incomplete and even fails on most lesions and MS patients due to failures in (1) OL progenitor cells (OPC) migration and differentiation into myelin-producing OLs, and/or (2) in myelin sheath production by surviving OLs (Franklin, 2002; Bacmeister et al., 2020). Being an expert in the area, Catherine Lubetzki highlighted, in 1996, the importance of neuron-OPC communication in the regulation of myelin formation. In her study, Lubetzki demonstrated that, while OLs can mature in culture without the presence of neurons, neuronal activity is required for the correct apposition of these myelin sheaths around neuronal axons (Demerens et al., 1996). These findings were further corroborated by Ragnhildur Káradóttir in a study, using an *in vivo* model, that showed the existence of glutamatergic synaptic transmission between OPC and demyelinated axons. Once the neuronal activity was blocked, her study demonstrated that OPC become “stuck” in a proliferative state without being able to receive the necessary cues to differentiate (Gautier et al., 2015). The same was recently proved in María Cecilia Angulo’s studies where recurrent stimulation of neuronal activity directly in lysophosphatidylcholine (LPC)-induced demyelinated axons, by photostimulation *in vivo*, increased local OL differentiation and led to the formation of new remyelinated myelin sheaths, followed by functionally recovery of neuronal activity in LPC-generated lesions (Ortiz et al., 2019). Additionally, Lubetzki’s lab, focusing on the mechanisms behind OPC differentiation into efficient myelinating cells, identified a novel promyelinating role of the ciliary neurotrophic factor, demonstrating that its incubation with myelinating co-cultures potentiated OL maturation and increased myelin formation *in vitro* (Stankoff et al., 2002). In another study led by Lubetzki, it was demonstrated that the polysialylated form of the neural cell adhesion molecule (PSA-NCAM), which is specifically expressed at the axonal surface during development to inhibit axonal attachment by OLs, is re-expressed on demyelinated axons within MS plaques. The same was not observed in matched control samples, hence unveiling a possible mechanism that can contribute to the inefficacy of axon re-ensheathment during MS progression (Charles et al., 2002). Furthermore, the same lab determined the relevance of netrin-1 on OPC recruitment to improve myelin formation. Indeed, netrin-1 is a guidance molecule known to repel OPC migration during neural development, and its expression can be found within MS lesions. Here, it is observed that netrin-1 production by astrocytes is potentiated upon induction of demyelination in MS lesions. The same early expression was not observed after experimental demyelination using LPC, a current model used to study efficient remyelination following demyelination. Therefore, Lubetzki’s studies reveal that temporal regulation of netrin-1 expression impacts remyelination. Particularly, they uncovered that netrin-1 overexpression at the onset of demyelination reduces the number of OPC arriving at the lesion site, and subsequently the number of mature/myelinating OLs (Tepavcevic et al., 2014).

Alongside, Yvonne Dombrowski, in a study led by Denise Fitzgerald, described a correlation between the presence of Treg cells and efficient remyelination. Indeed, Treg-depleted animals subjected to LPC treatment had decreased numbers of mature OLs and remyelinated axons. On the other hand, Treg administration to mice was able to rescue impaired OL differentiation and significantly increase the amount of fully mature OLs in both LPC- and cuprizone (CPZ)-induced lesions. When incubating Tregs, and particularly the Treg-derived regulatory protein CCN3, with neonatal murine *ex vivo* brain explants, the authors were able to observe an increased myelination index, confirming Tregs pro-myelinating capacity, and unveiling CCN3 protein as the mediator of Treg-driven effects on OPC differentiation and CNS myelination (Dombrowski et al., 2017). Furthermore, Xin Xie’s group also interested in searching for potential molecules that could modulate OPC differentiation, found L-ascorbyl-2-phosphate (As-2P, a stable form of Vitamin C), as a candidate for promoting OL differentiation and maturation *in vitro*. Here, As-2P promoted gradual expression of the OL lineage markers O4, CNPase, and MBP, facilitating the formation of myelin sheaths in myelinating co-cultures, and effectively promoted efficient remyelination *in vivo* following CPZ-induced demyelination (Guo et al., 2018). Moreover, Regine Sitruk-Ware’s group addressed the potential use of progesterone and the synthetic 19-nor-progesterone derivative nestorone as remyelinating therapeutic agents (El-Etr et al., 2015). Several studies have already reported their beneficial effects in *ex vivo* cerebellar cultures (Ghoumari et al., 2003), LPC models of demyelination (Hussain et al., 2011), and EAE mice (Giatti et al., 2012). Here, using the CPZ model, Sitruk-Ware’s study demonstrated that in progesterone-treated mice, MBP expression loss was reverted to levels comparable to those observed in controls, and the number of proliferative OPC and mature OLs were increased, which led to an augmented myelin recovery in the corpus callosum and cerebral cortex. Relevantly, the effects were recapitulated when administering nestorone (El-Etr et al., 2015). Later, in 2021, the same group demonstrated that nestorone alone or in combination with estradiol was able to increase levels of three transcription factors involved in myelin production, the Myt1, Olig2, and Sox17, offering new perspectives for the use of synthetic progestogens in myelin recovery therapies for long-lasting demyelination (El-Etr et al., 2021).

Another significant breakthrough in remyelination-based research was the recognition of astrocytes and microglia as critical participants in remyelination. In recent years, astrocytes have attracted a great level of attention in the study of demyelinating disorders. Particularly, Cheryl F. Dreyfus’s findings have contributed to the rising knowledge on the astrocytic contribution to myelin recovery in MS. Indeed, Fulmer et al. (2014) showed that stimulation of BDNF-release from astrocytes following CPZ-induced demyelination, using a single injection of a metabotropic glutamate receptor agonist, was able to restore the observed loss of myelin-associated proteins in the demyelinated corpus callosum. Later, in 2020, Dreyfus’ lab further demonstrated that by promoting a continuous production of astrocytic-derived BDNF, starting 4 weeks after CPZ treatment, they were able to increase the number of OLs, the number of myelinated axons and myelin thickness surrounding the axons. Relevantly, the observed results on myelin recovery were accompanied by the attenuation of the CPZ-associated behavioral deficits (Saitta et al., 2021). Finally, in another study, Feliu et al. (2017) were also able to correlate the production of chondroitin sulfate proteoglycans (CSPGs) by the reactive astroglial scar with axonal loss and impaired remyelination. In her study, the CSPGs’ production was therapeutically reduced around demyelinated areas of Theiler’s murine encephalomyelitis virus (TMEV)-infected mice, which led to a higher number of mature OLs, as well as to an increased number of myelinated axons with thinner myelin sheaths (indicators of remyelination) in the spinal cord of treated animals. Again, the increased remyelination efficiency was accompanied by an improvement in the TMEV-associated motor activity deficits, therefore unveiling the astrocytic potential as targets for MS recovery (Feliu et al., 2017). Together with astrocytes, microglial cells are also seen as major components in the process of myelin re-ensheathment post-CNS demyelination. Indeed, María Domercq’s study showed that the purinergic receptor P2×4R in microglia was involved in the modulation of microglial inflammatory responses and remyelination. By blocking P2×4R signaling at 10 days post-EAE induction, they observed activation of microglial pro-inflammatory pathways and inhibition of myelin debris phagocytosis by microglia. However, when potentiating P2×4R signaling, they were able to induce a microglial switch to a more anti-inflammatory phenotype, increasing myelin debris clearance, and remyelination, which consequently led to an ameliorated EAE disease outcome (Zabala et al., 2018). Alongside, Stella Tsirka’s lab, by depleting most of the inflammatory microglia and macrophages during the symptomatic stage of EAE, noticed a significant decrease in demyelinated WM areas, an increase in the number of mature OLs and an amelioration of the EAE-related behavioral deficits (Nissen et al., 2018). Later, in 2019, Amy Lloyd, in a study led by Veronique Miron, also demonstrated that for efficient remyelination to occur, a significant death of pro-inflammatory microglia must occur, allowing an efficient repopulation of new microglia with pro-regenerative properties (Lloyd et al., 2019). Interestingly, in the same year, Claudia Verderio’s group demonstrated that EVs produced by pro-regenerative microglia, when injected into LPC-induced demyelinated lesions, were able to boost myelin repair. On the contrary, injection of EVs from inflammatory microglia clearly blocked OPC maturation and remyelination in LPC-treated mice. Relevantly, it was observed that the inhibitory effect on OPCs was dependent on the presence of astrocytes, once again emphasizing the participation of astrocytes on lesion recovery mechanisms following demyelination (Lombardi et al., 2019). Finally, in a recently published study led by Anne Desmazières, it was observed that microglia directly interact, dependent on neuronal activity, with the nodes of Ranvier and that this interaction also regulates the phenotype of microglia. By blocking potassium flux between microglia-node interaction, not only there was a reduction in the percentage of nodes contacted by microglia, but it also induced a reduction in the expression of pro-regenerative markers, which was associated with a decreased percentage in the area of remyelinated axons in LPC-demyelinated cerebellar slices (Ronzano et al., 2021).

However, microglia have also been implicated in MS recovery through their capacity to clear fragmented myelin debris (Lampron et al., 2015). Indeed, Francesca Cignarella’s work, led by Laura Piccio, demonstrated that myelin-enriched microglia in active demyelinating CNS lesions of MS patients have increased expression of the triggering receptor expressed on myeloid cells-2 (Trem-2), a receptor associated with the microglial phagocytic pathway. In this study, the authors correlated the upregulation of Trem2 with the increased efficiency of microglia to clear myelin debris. Whereas Trem2 haploinsufficient mice reduced microglial capacity to remove myelin debris after 4 weeks of CPZ treatment, treating mice with a Trem2 agonistic antibody, accelerated the clearance of myelin debris by microglia, their intracellular degradation, and contributed to an increase in the density and differentiation of OLs in areas of demyelination with a subsequent enhancement of new myelin formation and axonal integrity (Cignarella et al., 2020). Nonetheless, although the presence of myelin-enriched microglia is a feature of MS active demyelinating lesions, the question of why remyelination fails in MS patients remains. To better elucidate the underlying mechanisms, we recently developed a probe, a modular fluorescent platform based on boronic acid salicylidenehydrazone complexes (BASHY), that is able to target myelin phagocytosing microglia in *ex vivo* cerebellar organotypic cultures and *in vivo* EAE cerebellum (Pinto et al., 2021). Also, in line with this and to further understand why remyelination fails in MS, Gesine Saher recently published a study with significant impact unveiling a link between the cholesterol metabolism in microglia and the process of myelin repair. Indeed, they demonstrated that in acute demyelinating conditions, microglia need an intact and functional cholesterol synthesis/metabolism pathway to promote remyelination. Microglia lacking sterol synthesis, as it was found during the acute phase of EAE and following CPZ- and LPC-mediated demyelination, exhibited a persistent inflammatory signature after phagocytosis of myelin debris. Conversely, exogenous prophylactical administration of squalene, a precursor of the sterol pathway, was able to decrease microgliosis, increase the density of mature OLs, enhance the myelinated areas after LPC demyelination, and ameliorate the EAE clinical score, therefore reinforcing the microglial support to remyelination, and emphasizing the involvement of microglial cholesterol metabolism pathway for novel therapeutic interventions in MS (Berghoff et al., 2021).

## 8. Conclusion

**FIGURE 1:**
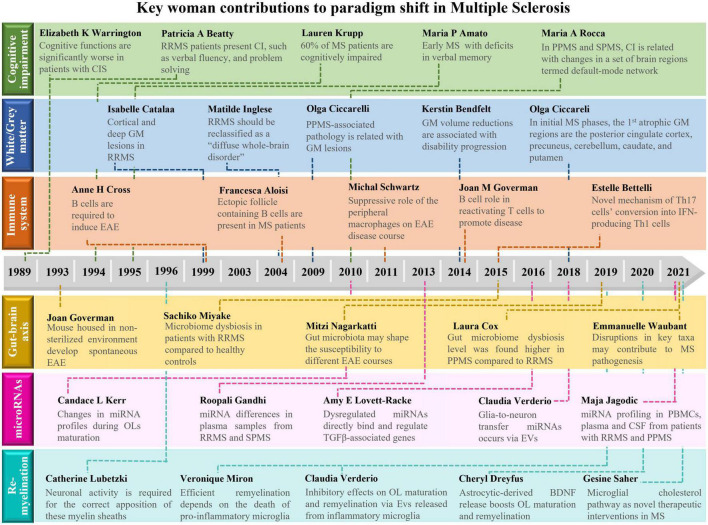
Schematic overview about the main contributions of women to shift paradigms in the field of Multiple Sclerosis. Here, we highlight the importance of discoveries made by women that shift paradigms in multiple sclerosis (MS) research. Major achievements were made on: (1) the definition of cognitive impairment as a major symptom in MS; (2) the impact of gray matter pathology on MS progression; as well as the relevance of (3) immune system cells, (4) gut microbiota, (5) miRNAs, and (6) remyelination in MS pathogenesis. BDNF, brain-derived neurotrophic factor; CI, cognitive impairment; CIS, clinically isolated syndrome; CSF, cerebrospinal fluid; EAE, experimental autoimmune encephalomyelitis; EVs, extracellular vesicles; GM, gray matter; IFN, interferon; miRNA, microRNA; MS, multiple sclerosis; OL, oligodendrocyte; PBMCs, peripheral blood mononuclear cells; PPMS, primary progressive multiple sclerosis; RRMS, relapsing-remitting multiple sclerosis; SPMS, secondary progressive multiple sclerosis; TGF, transforming growth factor.

Findings related with MS pathology are mainly attributed to men. This is due, at least in part, to the numerous obstacles that were posed to women in accessing higher education, since this path and the following scientific research would prevent their predisposed role: take care of husband, children, older people, and housework. Furthermore, women still represent just 33.3% of researchers globally, and their work rarely gains the recognition it deserves, with less than 4% of Nobel Prizes for science awarded to women and only 11% of senior research roles held by women in Europe. So, this review emphasizes the excellent work and devotion of many leading women researchers throughout the years, that has undoubtedly contributed enormously and significantly to break several paradigms associated with MS pathology (Figure 1). Although we have performed an extensive revision of the literature, we apologize to those colleagues whose interesting works have not been cited or recognized. However, by enhancing the recognition for the scientific contributions of all cited senior woman researchers, we truly believe that we are inspiring, engaging, and opening new possibilities of studies to encourage other women to fulfill the missing dots.

## Author contributions

AF proposed the literature review topic. AB, CB, AA, AR, and MVP drafted the manuscript. AB, CB, and AF revised the manuscript. All authors contributed to the manuscript and approved the submitted version.
